# Supplementary figures and images for: The dynamics of centromere assembly and disassembly during quiescence
Source: J Cell Biol. 2026 Apr 9;225(6):e202509067. doi: 10.1083/jcb.202509067 (PMC13064892; doi:10.1083/jcb.202509067)

F1K, CENPT

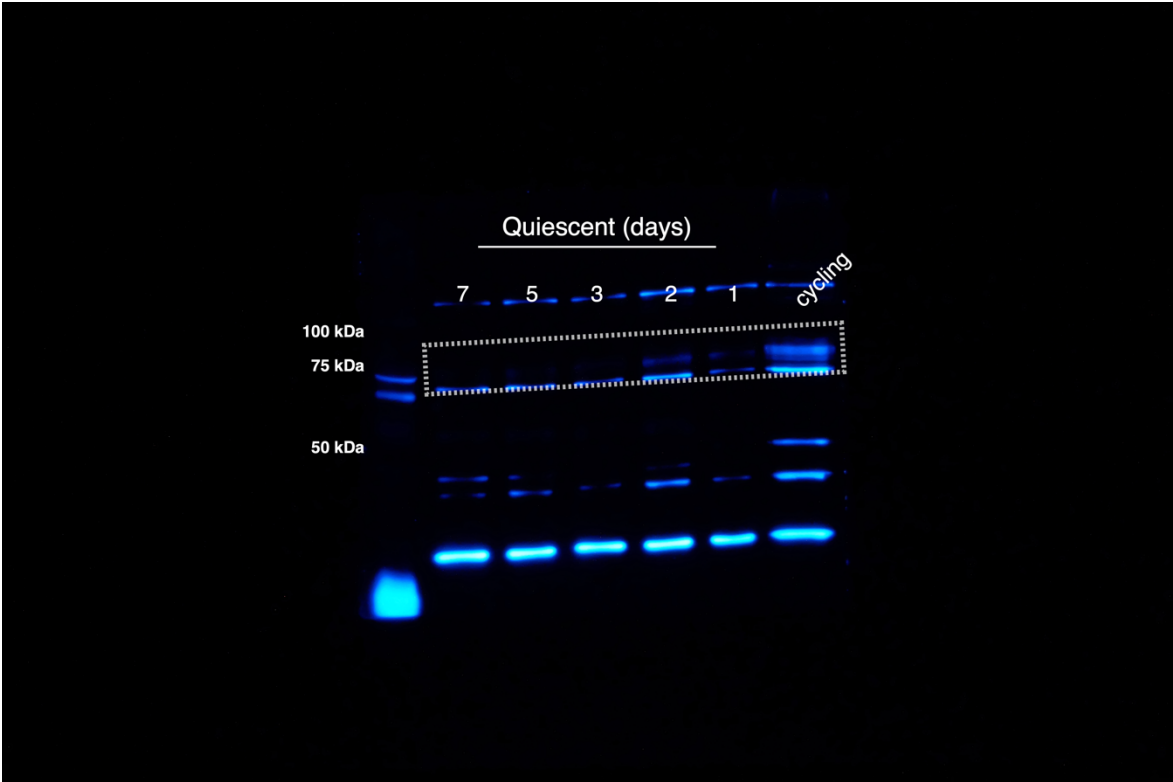

F1K, actin

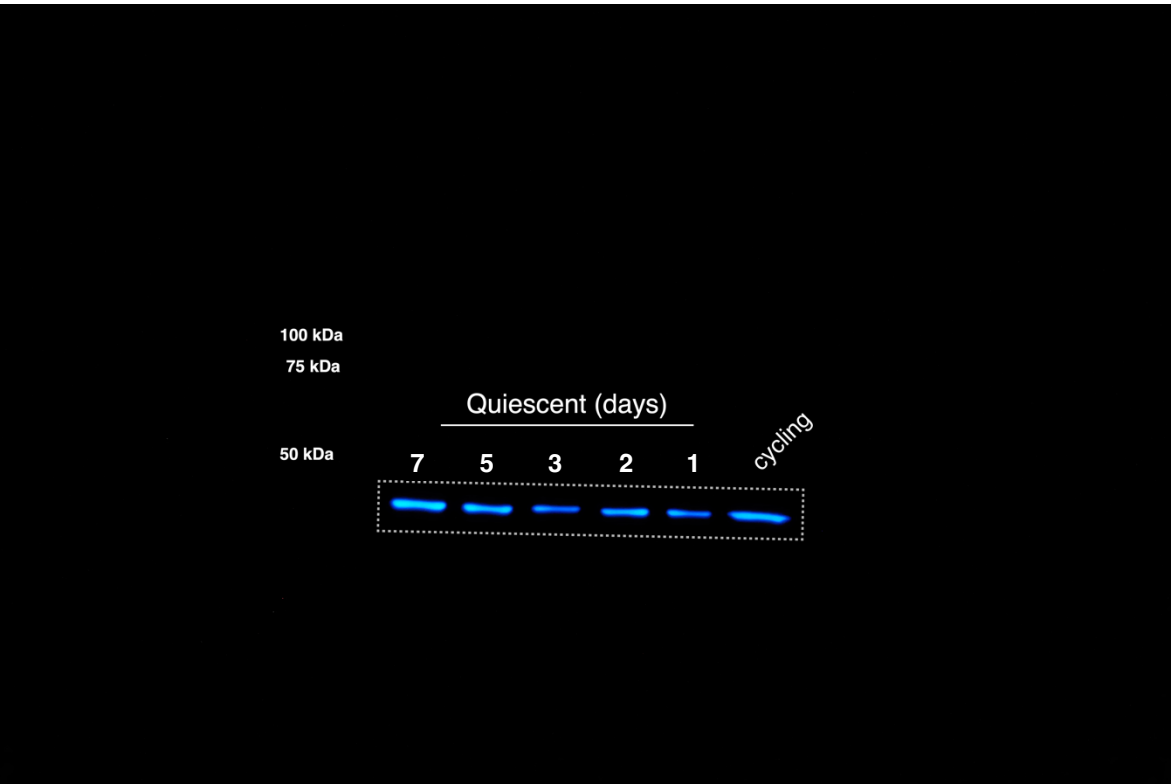

F1K, CENPH

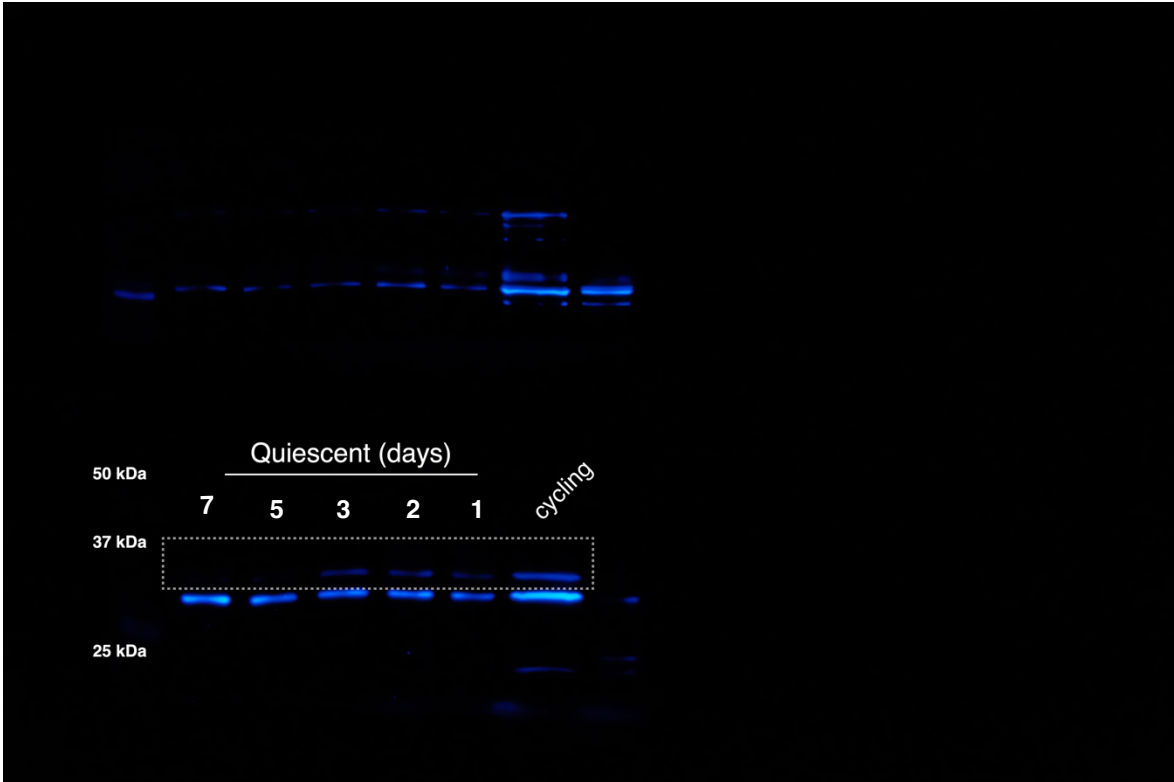

F1K, actin

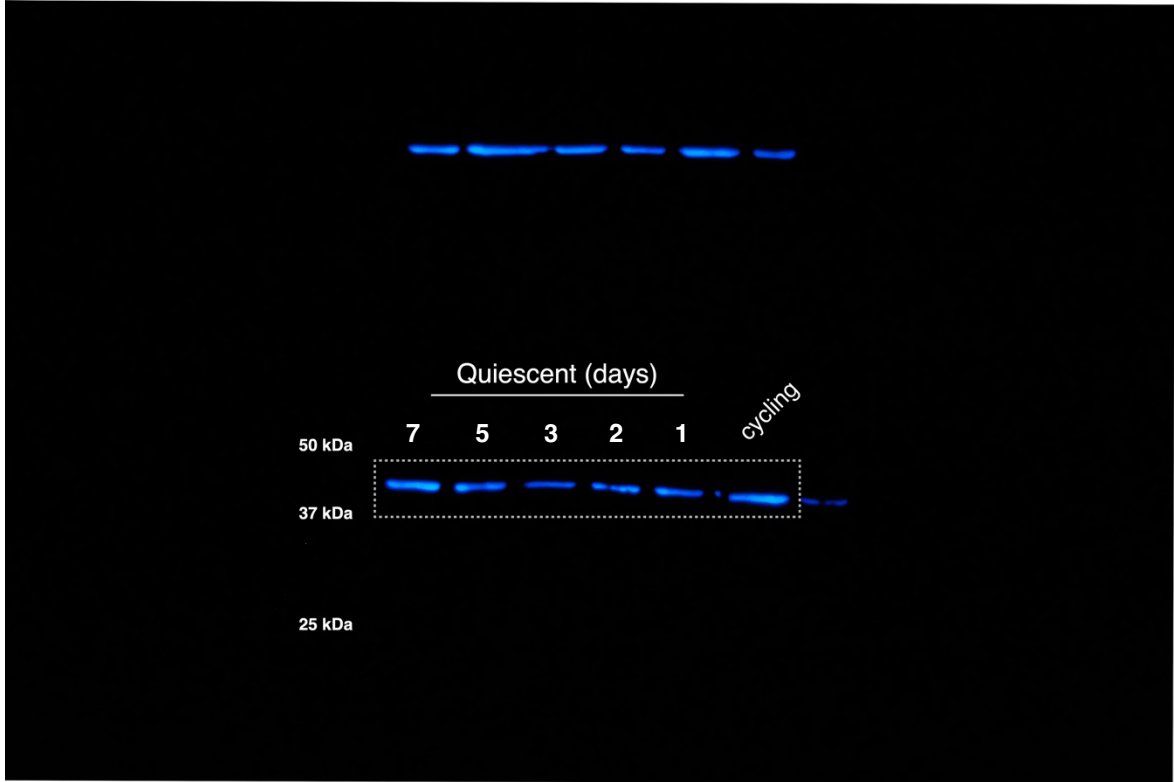

F1L, CENPC

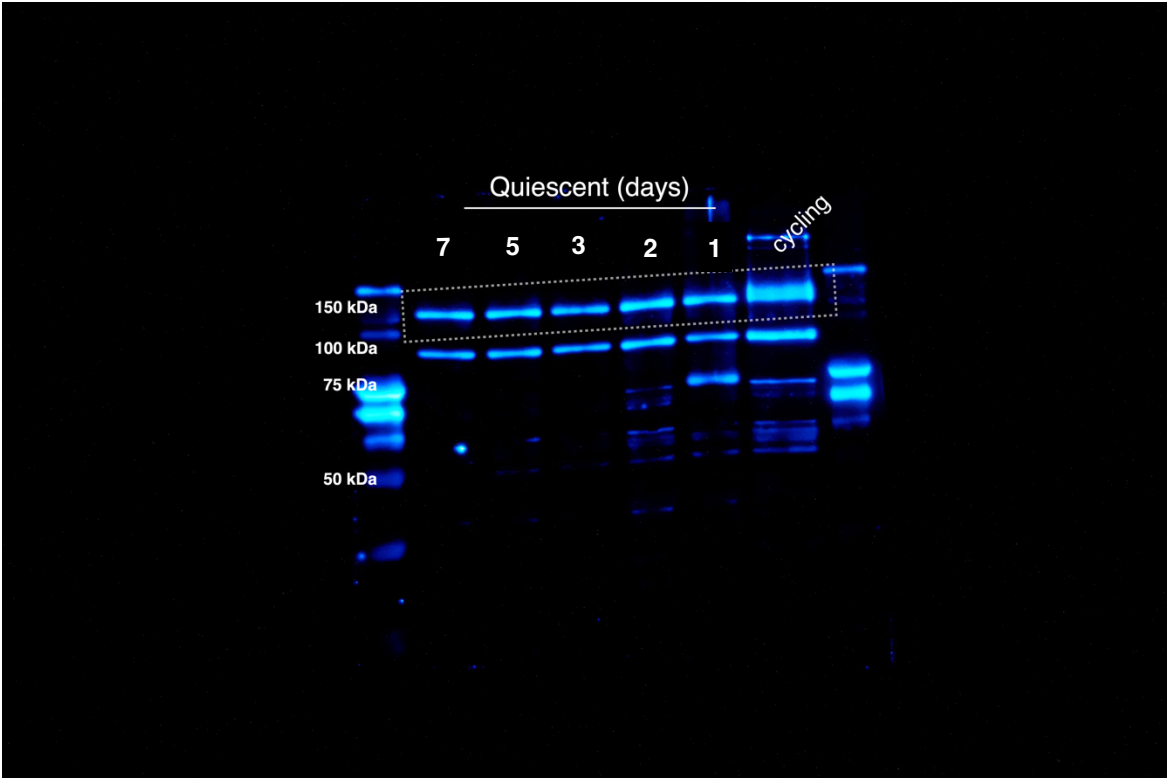

F1L, actin

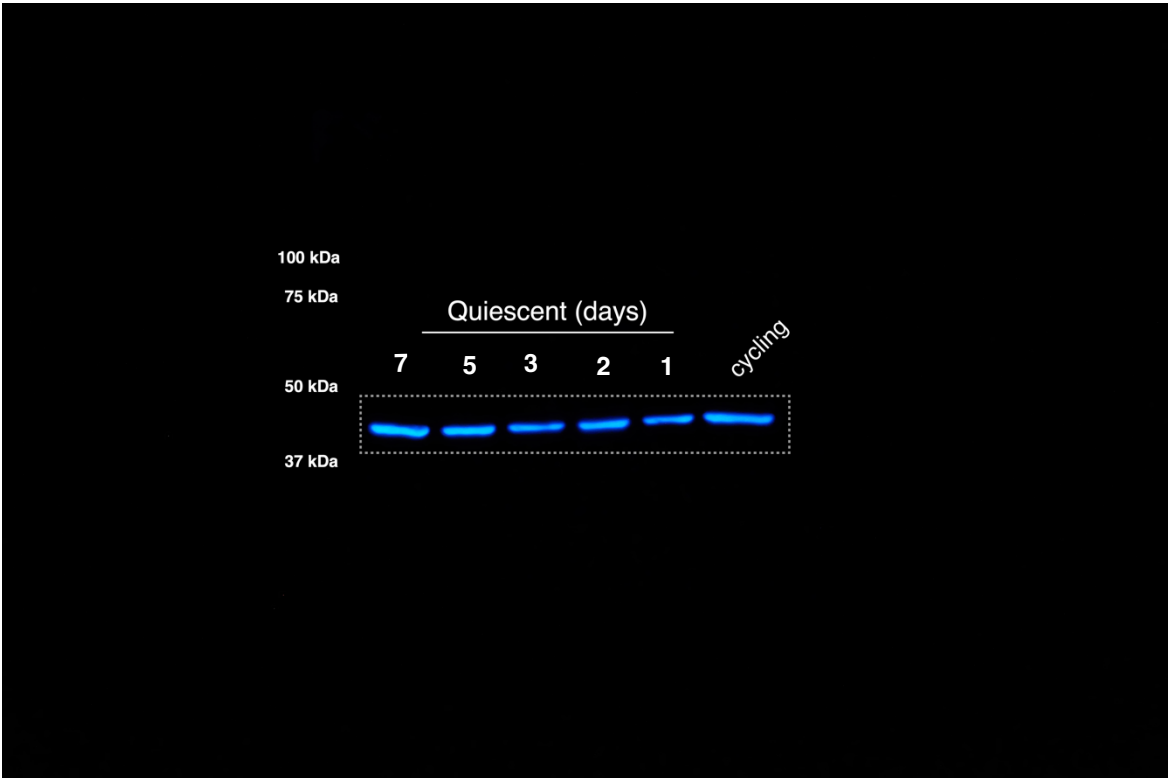

Supplement: SourceData F1 — is the source file for Fig. 1. [file jcb_202509067_sourcedataf1.pdf]

### F3G, cyclin B1

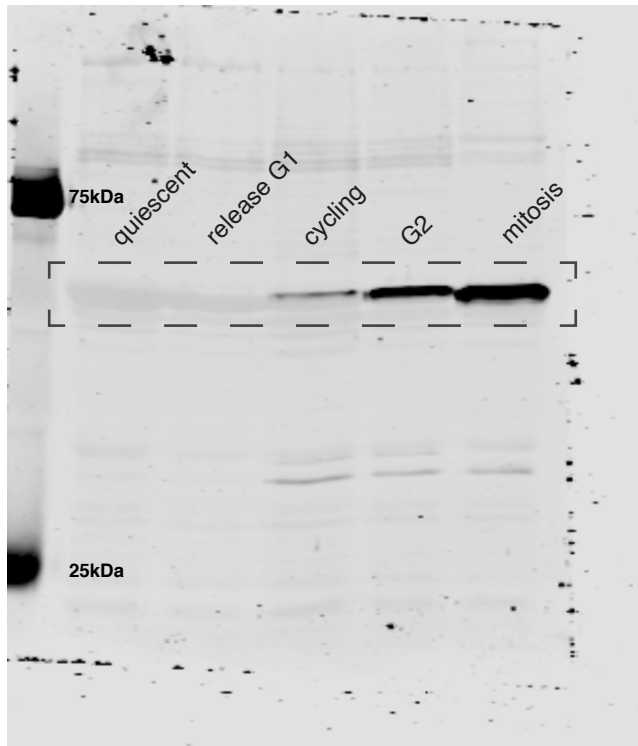

### F3G, tubulin

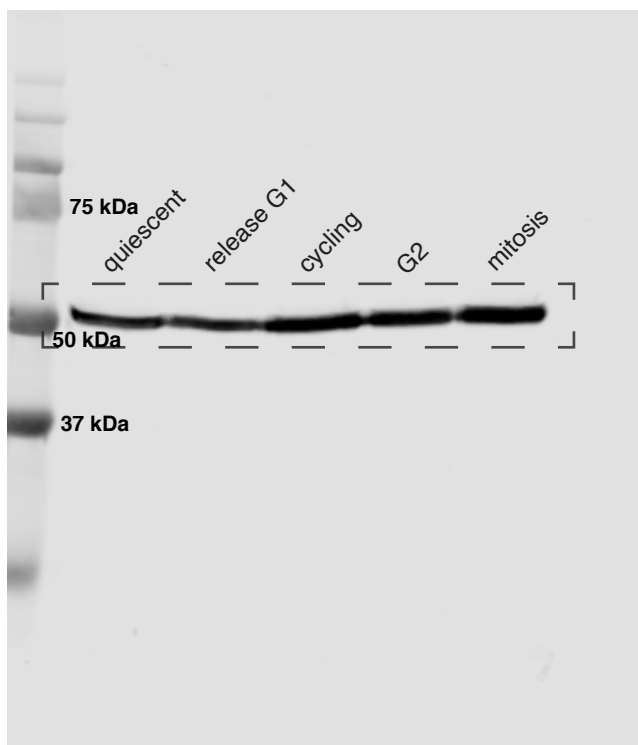

Supplement: SourceData F3 — is the source file for Fig. 3. [file jcb_202509067_sourcedataf3.pdf]

## FS1I, CENPT

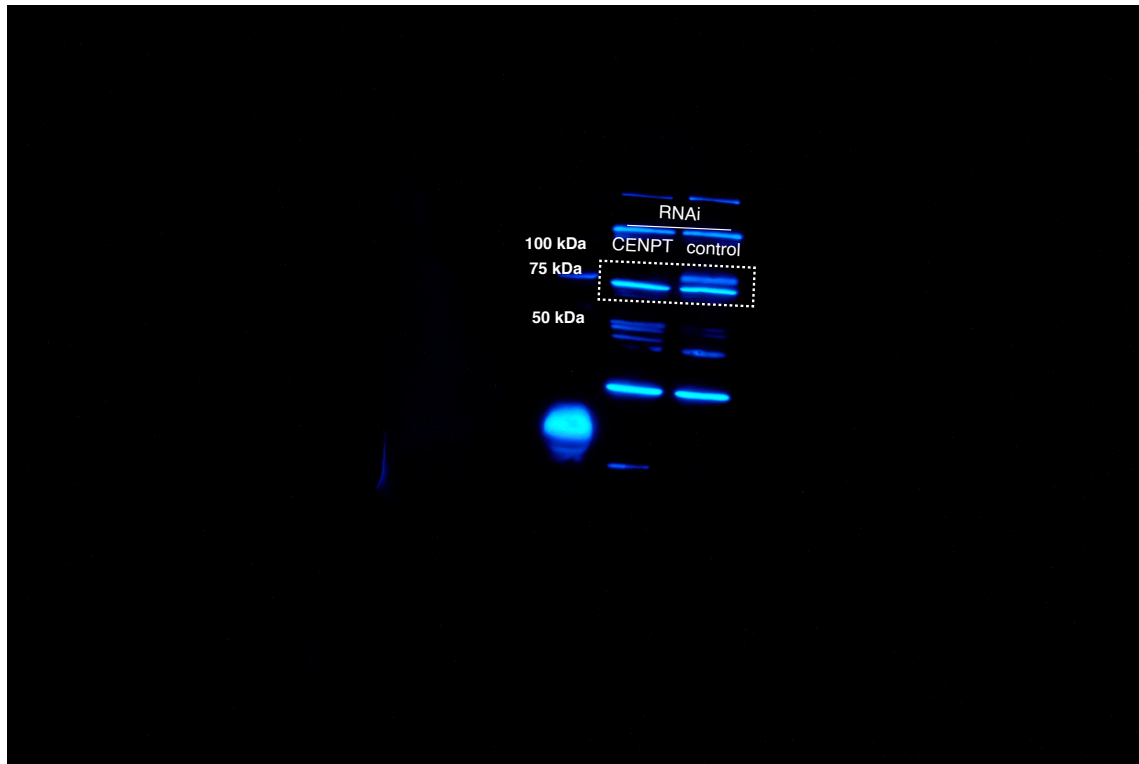

## FS1J, CENPC

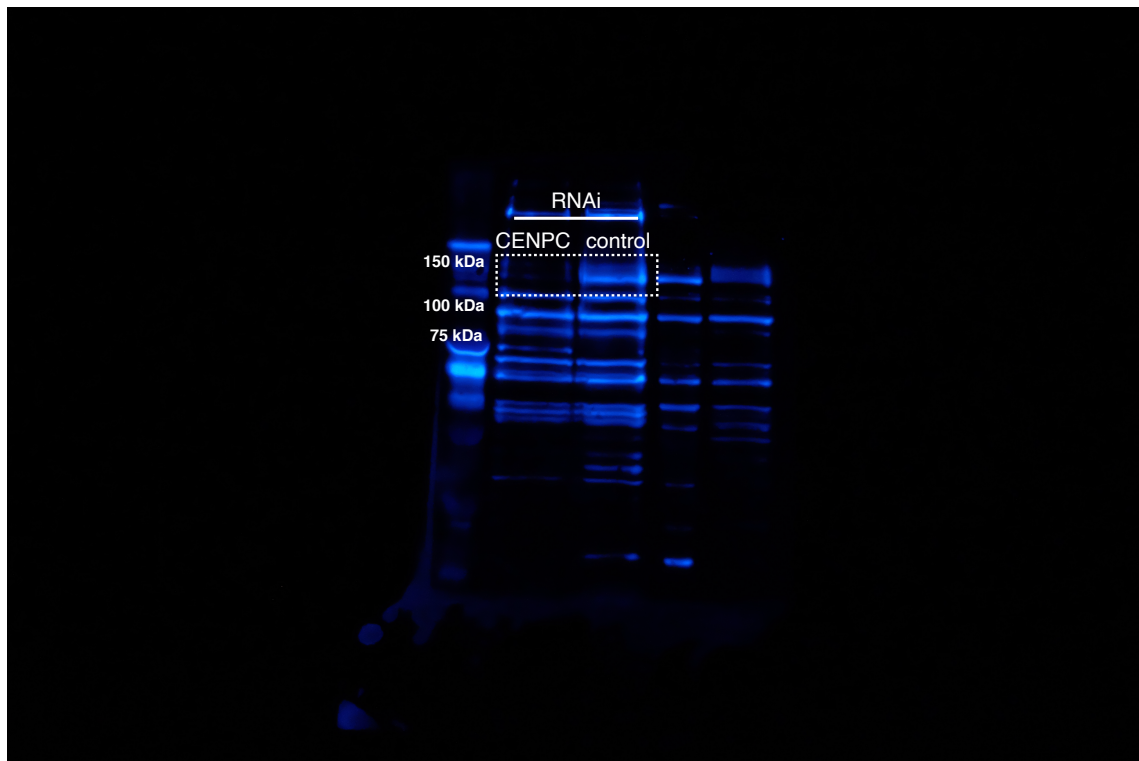

## FS1K, CENPH

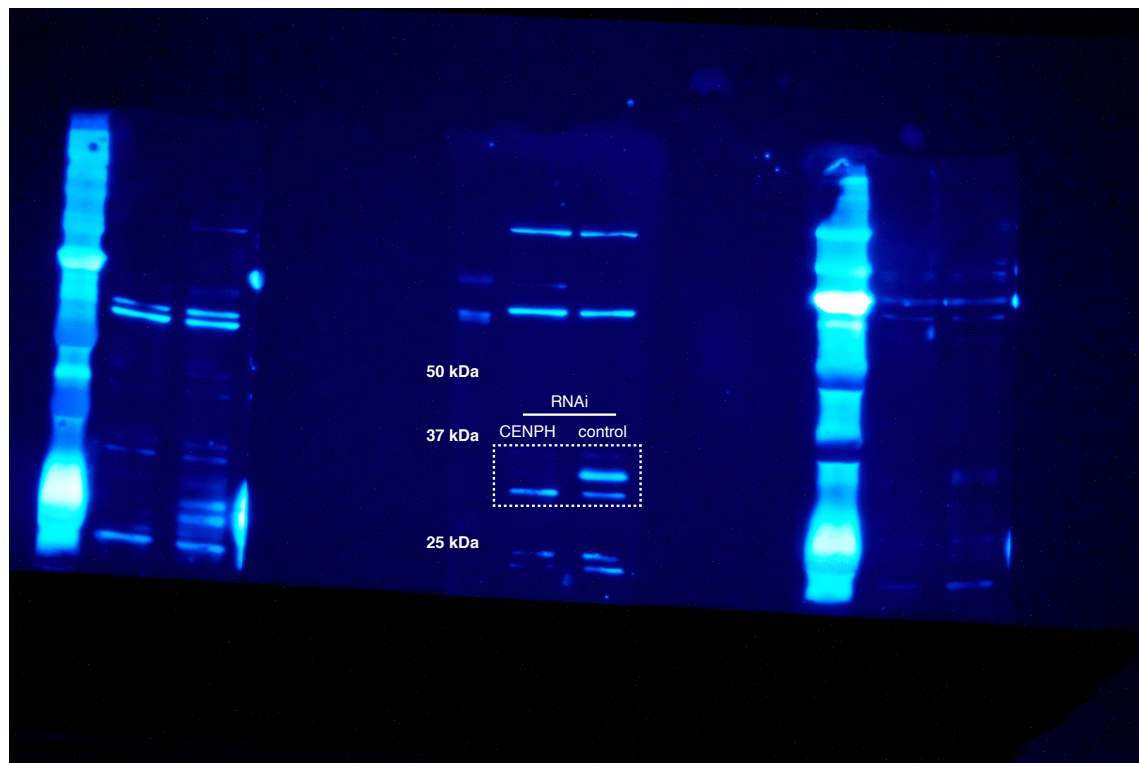

Supplement: SourceData FS1 — is the source file for Fig. S1. [file jcb_202509067_sourcedatafs1.pdf]

FS3H, CENPT, CENPH

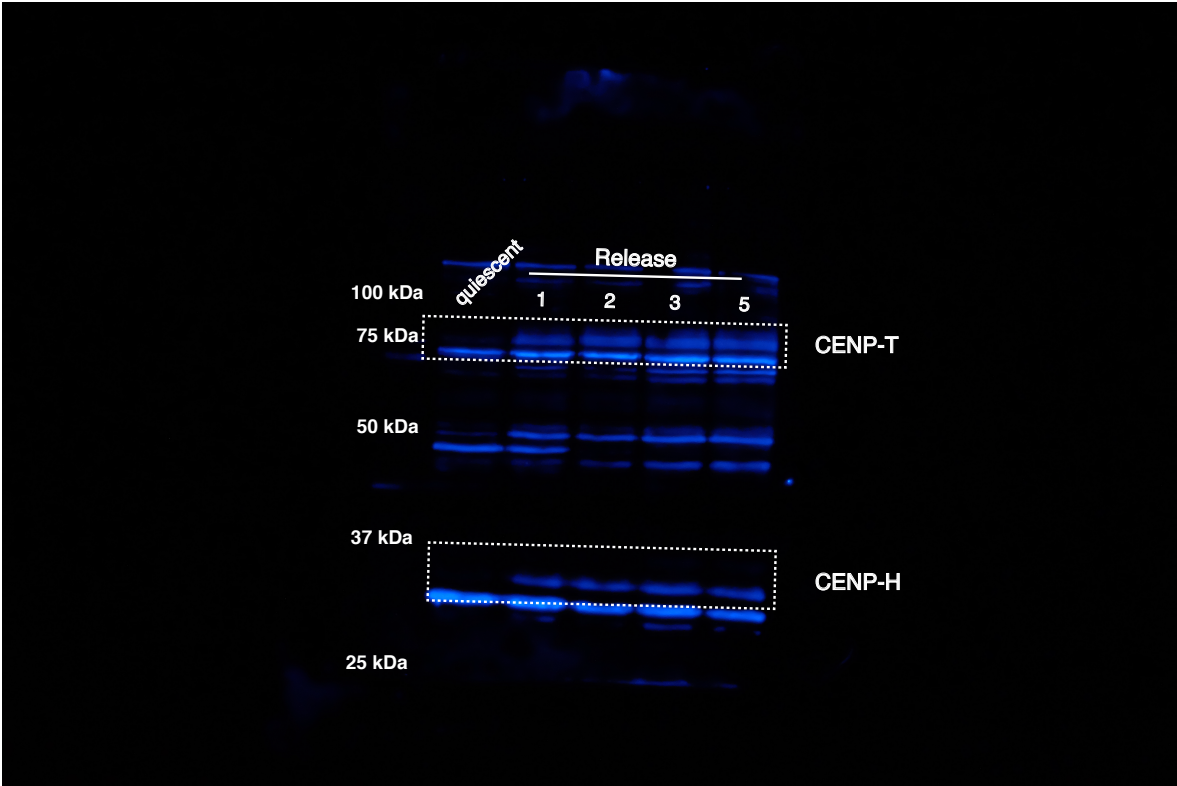

FS3H, actin

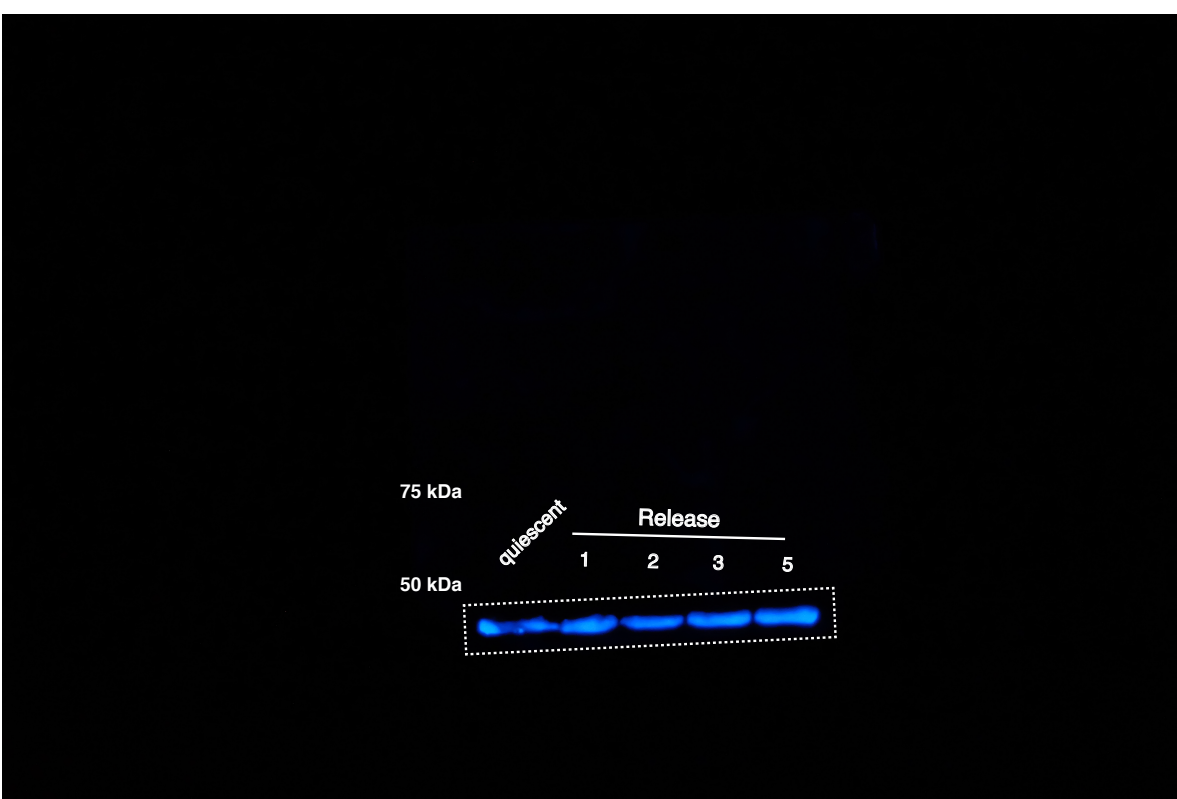

## FS30, CENPC, CENPH

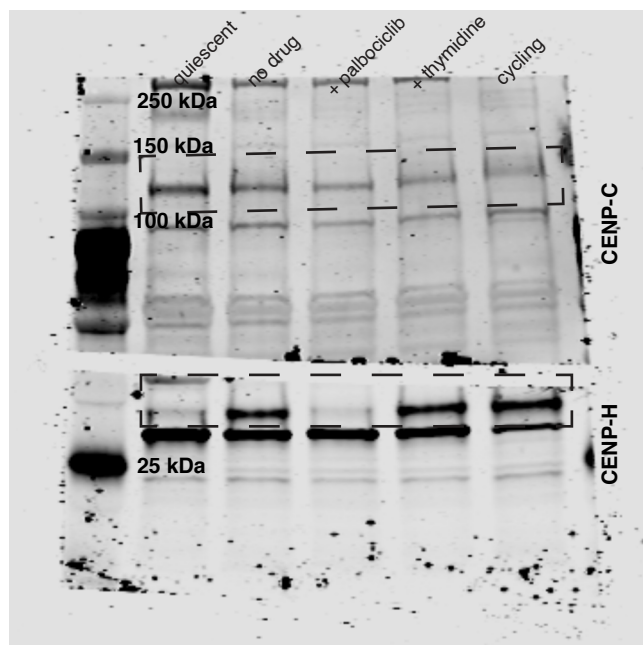

## FS30, CENPT

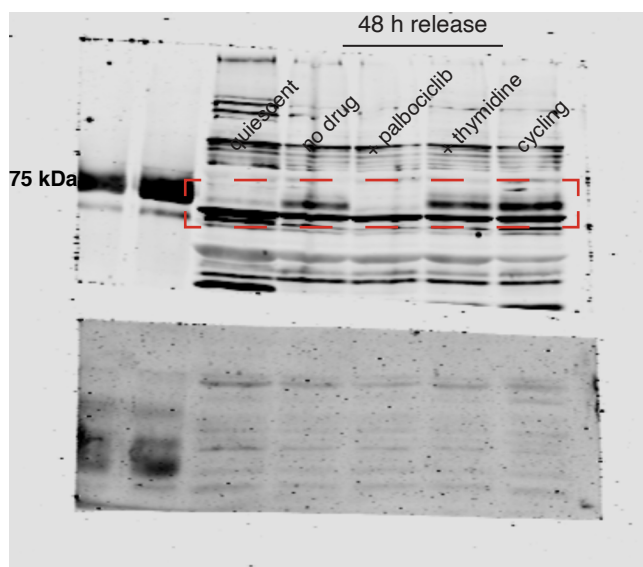

## FS30, tubulin

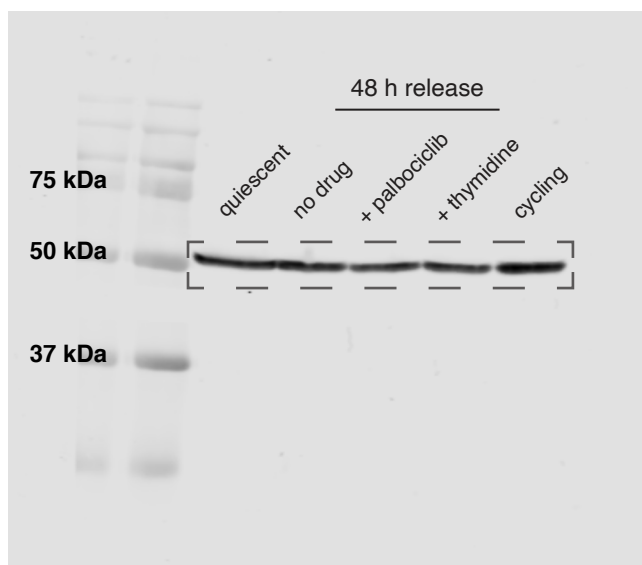

Supplement: SourceData FS3 — is the source file for Fig. S3. [file jcb_202509067_sourcedatafs3.pdf]
